# Connaissances et perceptions de la malnutrition par la population rurale des Hautes Terres Centrales, Madagascar

**DOI:** 10.11604/pamj.2021.39.277.21568

**Published:** 2021-08-27

**Authors:** Lantonirina Ravaoarisoa, Mamy Jean Jacques Razafimahatratra, Hanitriniaina Rasolofozafy, Dolorès Pourette, Jean De Dieu Marie Rakotomanga, Julio Rakotonirina

**Affiliations:** 1 Institut National de Santé Publique et Communautaire, Antananarivo, Madagascar,; 2 Institut de Recherche pour le Développement, Paris, France,; 3 Faculté de Médecine d´Antananarivo, Antananarivo, Madagascar

**Keywords:** Connaissances, malnutrition, Madagascar, perception, Knowledge, malnutrition, Madagascar, perception

## Abstract

**Introduction:**

la malnutrition par insuffisance d´apport alimentaire reste un défi majeur des pays à faible et moyen revenu. La présente étude vise à identifier les constructions socioculturelles qui interviennent dans les présentations de la malnutrition.

**Méthodes:**

une étude qualitative a été réalisée dans la région Amoron´i Mania, Madagascar. Les participants sont constitués par les femmes enceintes, les mères et pères de famille, les grand-mères ainsi que les acteurs de la santé comme les matrones, les agents communautaires et les agents de santé. Au total, 24 entretiens individuels semi-directifs et 6 focus groups ont été réalisés pour collecter les données. L´analyse thématique a été utilisée.

**Résultats:**

la malnutrition renvoie à une idée de manque d´aliments et de sous-alimentation. Elle tourne autour de la consommation quantitative du riz, des faits socioculturels et des problèmes de ressources financières. Les groupes vulnérables étaient principalement les enfants et les femmes enceintes. La perception de la malnutrition avec ses signes était grave, mais c´est une situation à laquelle la population a fini par s´adapter. Ainsi, des moyens ont été mis en œuvre par les familles pour lutter contre la malnutrition.

**Conclusion:**

le contexte socioculturel module les connaissances et la perception sur les causes, les manifestations et le caractère vulnérable ou non d´un individu ainsi que sur la gravité de la malnutrition.

## Abstract (en)

**Introduction:**

malnutrition due to inadequate food supply is a major challenge in low- and middle-income countries. The purpose of this study is to identify the sociocultural drivers of malnutrition.

**Methods:**

we conducted a qualitative study in the Amoron´I Mania region, Madagascar. The study involved pregnant women, mothers and fathers, grandmothers and health actors such as “matrones”, community workers and health workers. A total of 24 semi-structured individual interviews and 6 focus groups were used to collect data. Thematic analysis was used.

**Results:**

malnutrition refers to a lack of food and undernourishment. It revolves around the amount of rice consumption, socio-cultural factors and insufficient financial resources. Vulnerable groups were mainly composed of children and pregnant women. Severe malnutrition including signs was reported, but there was evidence for local adaptation. Thus, families were trying by different means to fight against malnutrition.

**Conclusion:**

the socio-cultural context modulates knowledge and perception of the causes, the manifestations and the vulnerability or non-vulnerability of an individual as well as the severity of malnutrition.

## Introduction

La malnutrition correspond aux carences, excès ou déséquilibres dans l´apport énergétique et/ou nutritionnel d´un individu [1]. Dans les pays à revenu élevé, les problèmes de surpoids, d´obésité ainsi que les pathologies médicales qui y sont liées, restent un défi majeur. Dans les pays à faible et moyen revenus, la principale préoccupation est plutôt la malnutrition par manque d´apports alimentaires [2]. Pour Madagascar, en 2013, 58% de la population étaient en situation d´insuffisance alimentaire [3] et le poids de la malnutrition sur l´économie du pays est estimé à 743 millions de dollars, soit 7% de son Produit Intérieur Brut (PIB). Ces pertes sont principalement liées à une perte de mains d´œuvre, une perte de productivités actuelle et future et une dépense liée au coût des soins de santé [4]. À part l´extrême pauvreté de la population (76% vit avec moins de 1,90 USD/jour) qui limite l´accès aux nourritures [5], d´autres facteurs déterminent le comportement alimentaire et pourraient entretenir la malnutrition [6]. La lutte contre la malnutrition se concrétise à Madagascar par la mise en œuvre des interventions inscrites dans le « Plan National d´Action pour la Nutrition » (PNAN) [7], regroupées selon trois axes principaux : la nutrition spécifique ciblant les causes immédiates de la malnutrition (alimentation inadéquate et maladies), la nutrition sensitive visant les facteurs sous-jacents (insécurité alimentaire, environnement insalubre et inaccessibilité aux soins) et la bonne gouvernance. Les principaux partenaires du pays dans la mise en œuvre de ce plan sont représentés par le Ministère de la Santé, les Agences des Nations Unies et les Organisations Non Gouvernementales (ONG). Malgré les efforts pour lutter contre la malnutrition, elle demeure un problème majeur pour le pays. La présente étude se propose d´identifier les constructions socioculturelles qui interviennent dans les représentations de la malnutrition et influencent l´adoption des comportements favorables à la lutte contre la malnutrition.

## Méthodes

### Sites d´étude

L´étude a été conduite dans la région Amoron´i Mania, une des 22 régions de Madagascar et située dans les Hautes Terres centrales. C´est une des régions les plus touchées par la malnutrition: 30,5% des ménages sont en situation d´insécurité alimentaire et 41,6% des femmes en âge de procréer sont en état de dénutrition (Indice de Masse Corporelle < 18,5 kg/m^2^) (3,8). L´étude a été menée en milieu rural, zone la plus frappée par la malnutrition [8].

### Type d´étude et populations cibles

Il s´agit d´une étude qualitative. La sélection des participants, tous âgés de plus de 18 ans, a été faite d´une manière rationnel et non aléatoire. Le choix s´est porté sur des individus qui jouent un rôle majeur dans le comportement alimentaire d´un ménage, des personnes qui peuvent avoir une influence particulière sur les autres membres de la famille et un groupe chez qui la fréquence du problème nutritionnel est rapportée comme élevée. À cet effet, des femmes enceintes et des mères de familles (chez qui la prévalence de la sous nutrition est élevée) [8], des grand-mères (jouant un rôle prépondérant dans l´alimentation des enfants) [9] et des pères de famille (maintenant généralement le rôle de chef de famille) ont participé à l´étude. Des individus œuvrant dans le domaine de la santé ont aussi été inclus: matrone (personne sans formation médicale, mais reconnue localement pour leur savoir-faire dans les soins entourant la grossesse, l´accouchement et la prise en charge des nouveau-nés) [10], agent communautaire et agent de santé. Le nombre d´individus à interroger dépendait de la saturation des informations obtenues lors de la phase de collecte. Le Tableau 1 récapitule le nombre et les caractéristiques des participants.

**Tableau 1:** caractéristiques des participants

Caractéristique	Femme enceinte (n=21)	Mère d´enfant < 5 ans (n=26)	Père de famille (n=4)	Grand-mère (n=4)	Matrone (n=4)	Agent commu nautaire (n=4)	Agent de santé* (n=4)
Age médian en année [Min-Max]	25 [18 - 38]	24 [19 - 41]	[23 - 40]	[45 - 68]	[49 - 62]	[40 - 53]	-
Profession							
Agricultrice (nb)	16	22	4	4	4	4	-
Epicière	2	1	_	_	_	_	-
Artisane	2	1	_	_	_	_	-
Institutrice	1	2	_	_	_	_	-
Situation matrimoniale (nb)							
Mariée	21	20	4	3	3	3	4
Non mariée	_	6	_	1	1	1	-
Niveau d´étude (nb)							
Illettrée	1	1	11	1	_	_	-
Primaire	11	10	_	2	2	_	-
Secondaire 1^er^ cycle	6	13	1	1	2	3	-
Secondaire 2^e^ cycle	3	2	2	_	_	1	-
Universitaire							4

* 3 sages-femmes et 1 infirmier

### Collecte et analyse des données

La collecte des données a été réalisée en octobre et novembre 2016. Elle a été assurée par des médecins spécialistes en santé publique mais ayant également d´autres spécialités (nutrition communautaire, socio-anthropologie, sciences sociales appliquées au développement). D´une part, des entretiens individuels approfondis ont eu lieu auprès des femmes enceintes, des mères de familles, des grand-mères, des pères de famille, des agents communautaires, des agents de santé et des tradipraticiens. Au final, après saturation des informations obtenues, 24 entretiens individuels de type semi-directif ont été effectués. D´autre part, 6 *focus groups* ont été réalisés: trois auprès d´un groupe de femmes enceintes, deux auprès des mères de familles non enceintes et un chez un groupe mixte composé de femmes enceintes et de mères de famille non enceintes. La taille de chaque entretien de groupe variait de 6 à 7 individus. Une même grille abordant la connaissance de la malnutrition et les moyens mis en œuvre pour y remédier a été utilisée chez les femmes enceintes, les mères, les grands-mères et les pères. Pour les agents de santé, les agents communautaires et les matrones, la grille d´entretien a été élaborée pour comprendre leur point de vue sur le problème de la malnutrition au sein de la communauté dans laquelle ils interviennent. Les entretiens (varient de 30 à 45 minutes et en dialecte local) ont été enregistrés dans leur intégralité, retranscrits et traduits en français et puis, analysés selon une approche thématique transversale.

### Considération éthique

Le consentement et l´accord des participants ont été obtenus verbalement après qu´une note d´information expliquant l´étude ainsi que leur droit, leur a été lu et donnée. Durant la phase d´analyse, seuls des individus mandatés avaient accès aux informations personnelles des participants. L´étude a obtenu l´accord du Comité d´Éthique du Ministère de la Santé Publique de Madagascar (Réf NÂ° 097 MSANP/CE du 25/11/13).

## Résultats

### Définition et causes de la malnutrition

Hormis les professionnels de santé et les agents communautaires, la malnutrition renvoie nos interviewés à une idée de sous-alimentation (« *tsy fahampian-tsakafo* » en malgache). La traduction de la malnutrition par l´Académie Malagasy (« *tsy fanjarian-tsakafo* ») n´est pas encore assimilée par la population. La consommation du riz comme aliment de base de la population, est prise comme référence pour définir la malnutrition. Considéré comme très nutritif, le consommer 3 fois par jour serait suffisant pour se protéger de la malnutrition. Lorsque les réserves en riz s´épuisent, les ménages se sentent comme plus vulnérables car la consommation s´oriente alors vers des produits jugés de moins bonne qualité (manioc, mais, patate, etc.).

*« La malnutrition, c´est parce que nous consommons du manioc, du maïs (...). Nous mangeons mieux durant la saison de récolte (...). Si d´habitude, je cuisine un et demi de kapoaka (3,5 kapoaka de riz équivaut à 1kg), c´est suffisant pour nous deux (...), pendant les saisons de pluie, nous ne pouvons pas faire pareil car un kapoaka de riz coûte 400 Ariary (soit 0,1 euro), ainsi on doit se contenter d´un kapoaka pour deux jours » (grand-mère, 68 ans)*.

Une mauvaise gestion du revenu, une inadéquation du revenu au nombre de bouches à nourrir ou le manque de volonté pour travailler ont également été mentionnés comme causes de la malnutrition. La priorisation de certaines obligations sociales et familiales comme le « *lagnonana* » (rite funéraire pratiqué dans certaines régions de Madagascar consistant à recouvrir de nouveaux linceuls les défunts, et occasion d´une grande festivité) et ce, au détriment des besoins alimentaires, a également été évoqué comme cause de la malnutrition.

*« Même s´ils ont de l´argent, (...) ils économisent pour payer la quote-part qu´ils devront payer pour le lagnonana (...) et serrent trop la ceinture » (Sage-femme, 45 ans)*.

### Les groupes vulnérables: les enfants, les femmes enceintes, les personnes âgées

En matière de malnutrition, les enfants en bas âge et les femmes enceintes seraient les plus vulnérables. Les personnes âgées (et aussi les femmes enceintes) le seraient du fait que souvent, elles sont incapables de travailler. Dans un contexte de grande pauvreté, les adultes se privent pour leurs enfants, et seraient souvent aussi touchés. Mais les hommes et les femmes ne sont pas impactés de la même manière. Le statut d´homme renvoie à l´activité et à la dépense en énergie. Même si dans les faits, les femmes participent à la survie de la famille, dans les représentations, elles sont davantage « passives » que les hommes et auraient donc des besoins alimentaires moindres. Cette représentation désavantage les femmes en matière d´alimentation. La culture locale légitime également un degré de proximité affective enfant-mère beaucoup plus marqué. Ainsi, les mères, déjà soumises aux tâches qui leur sont socialement attribuées, sont aussi les premières à se sacrifier pour leurs enfants.

*« Les enfants et les hommes doivent manger, il faut les aider car ils supportent moins la faim. Les mères doivent céder. C´est ce qui fait que beaucoup des femmes sont atteintes de malnutrition par ici (...). Dernièrement, j´ai fait accoucher une femme mère de 15 enfants. Et là, à chaque fois que le mari sert à manger à la mère, cette dernière partage sa nourriture avec leurs enfants » (Matrone, 62 ans)*.

Pour les femmes enceintes, le régime devrait être diversifié et composé des produits considérés comme riches en « vitamine » (comme les poissons, les fruits, les œufs, etc.). L´accès à ces produits est pourtant considéré comme difficile à cause de la pauvreté ou parce que le produit n´est pas disponible localement.

*« La principale cause de la malnutrition des mères est la difficulté financière, parce que si l´argent manque, on ne peut pas se permettre d´acheter des nourritures diversifiées » (Focus group, Femmes enceintes)*.

La question de tabous et d´interdits alimentaires jouent également un rôle. Certaines femmes enceintes éviteraient certains aliments pour éviter certaines maladies.

*« Il y a certaines coutumes, certains tabous qu´on essaie d´expliquer aux femmes (...) C´est le cas du poisson, par exemple: des femmes enceintes n´en mangent pas de peur que cela puisse provoquer le « Farasisa » (une sorte de maladie de la peau) (Agent de santé)*.

### Les signes de la malnutrition (Tableau 2)

**Tableau 2:** résumé des résultats de l’étude

Connaissances sur la malnutrition	Perceptions de la malnutrition
- Malnutrition: un état de sous-alimentation, d’insuffisance en apport alimentaire - Personnes vulnérables: les enfants, les femmes enceintes et les personnes âgées - Causée par une vulnérabilité individuelle/condition physique et par la pauvreté - Symptômes: maigreur, faiblesse et mauvaise mine.	- Situation grave nécessitant une solution urgente. Elle est grave car expose la personne à des maladies et aussi provoque des conséquences sur le plan social. - Apanage des gens pauvres, de certains groupes d´individus (les groupes vulnérables) - Le riz en tant qu´aliment de base, est perçu positivement comme protecteur contre la malnutrition. Ne pas en consommer suffisamment expose au risque de malnutrition.

L´état d´une personne malnutrie se rapprocherait de l´état d´une personne malade auquel on associe souvent faiblesse, maigreur et mauvaise mine.

*« Une personne malnutrie est fatiguée et maigre, elle a une mine toute différente: comme le mien en ce moment car je viens d´être malade et je suis fatiguée; j´ai le visage fatigué et pâle, avec beaucoup de rides » (Grand-mère, 68 ans)*.

Les symptômes et les apparences ne sont pas les seuls pouvant aider à identifier un individu malnutri. Des situations d´aspects sociaux ou médicaux comme les échecs scolaires et les accouchements difficiles, seraient aussi liées à la malnutrition. Par ailleurs, la pauvreté financière d´un ménage pourrait suffire pour dire que ses membres sont en situation de malnutrition.

*« On connait celles ou ceux qui ont beaucoup d´enfants, celles ou ceux qui ont des difficultés, qui n´ont pas suffisamment de terre (...) » (Matrone, 49 ans)*.

### Perception de l´importance et de la gravité du problème de malnutrition (Tableau 2)

La malnutrition est considérée comme un problème grave. Elle serait une maladie grave en elle-même, rendant les gens vulnérables aux maladies et ayant des conséquences sur le plan social. Une mère affaiblie par la malnutrition ne pourrait assurer ses rôles envers sa famille et ses enfants.

*« C´est vraiment un problème majeur parce que tout dépend de la mère, donc, si la mère est malnutrie, rien ne va à la maison, elle n´arrive même pas à balayer la maison » (Père, 40 ans)*.

Les agents de santé et les agents communautaires ont toutefois affirmé que dans les faits, la population a fini par s´adapter à leur situation de malnutrition et elle ne la considère plus comme situation d´urgence.

*« La malnutrition engendre des conséquences sur la vie familiale (...) mais les gens ne la considèrent plus comme un problème » (Sage-femme, 45 ans)*.

### Les actions et moyens mis en œuvre au niveau individuel et au sein du ménage pour lutter contre la malnutrition

Se disant conscients de leur vulnérabilité face à la malnutrition, les interviewés affirment avoir fait des efforts pour changer les choses. Les stratégies sont multiples: améliorer l´agriculture et l´élevage, mobiliser tous les potentiels humains du ménage qui peuvent travailler, gérer la consommation et le panier alimentaire.

## Discussion

La malnutrition est un état résultant d´une alimentation inadéquate ou insuffisante, mais les mots utilisés par la population pour désigner ces deux situations sont identiques : « tsy ampy sakafo ». D´où une confusion entre la définition de « malnutrition » et d´ « insuffisance alimentaire ». Comme toute pratique, les pratiques alimentaires se réfèrent à un cadre socioculturel et à certaines traditions [11]. Chez les malgaches, la consommation journalière du riz constitue un symbole de la perpétuation de la culture [12]. Le riz y est considéré comme un produit essentiel, faisant d´un repas, un vrai repas [13] et tout problème au niveau de sa disponibilité engendre un sentiment de vulnérabilité à la malnutrition. Une étude conduite chez des réfugiés vivant dans un camp en Mauritanie, a trouvé que la malnutrition s´explique par l´effet du changement brusque de leur cadre de vie qui les ont obligés à consommer d´aliments qui leur sont inhabituels [14].

Dans les pays à faible revenu, la principale cause de la malnutrition reste la difficulté financière [15]. Pour la région Amoron´i Mania, le taux de pauvreté y est supérieur à la moyenne nationale [16]. Cette pauvreté s´insère dans un contexte culturel particulier. Les malgaches sont, en effet, un peuple connu pour son attachement à sa culture, aux âmes et au monde surnaturel [17]. Sur les Hautes Terres, la pratique du « famadihana » ou « milagnona » en est l´une des expressions. L´organisation d´un tel événement est pourtant très coûteuse et les revenus des ménages, déjà très faibles, se trouvent encore amputés des quotes-parts qu´ils doivent verser pour les préparatifs. Ce contexte culturel vient surement s´ajouter aux facteurs de vulnérabilité individuelle.

Pour nos interviewés, les enfants et les femmes enceintes et allaitantes sont les principaux groupes qui nécessiteraient un apport alimentaire particulier. Cette perception n´est pas contraire aux différentes observations scientifiques [18]. Toutefois, la difficulté financière demeure un frein majeur dans l´accessibilité aux produits nécessaires.

Dans les représentations, il serait aussi attendu des femmes enceintes qu´elles ne travaillent pas ou du moins, réduisent leurs activités physiques. Dans la réalité pourtant, les femmes enceintes travaillent presque jusqu´à l´accouchement et ce n´est qu´après seulement qu´elles restent confinées chez elles, inactives et bénéficient de tous les grands soins des membres de leur famille [10]. Durant cette période dite de « mifana », elles sont particulièrement gâtées en matière d´alimentation.

La population de la région tire généralement ses revenus des activités consommant fortement de l´énergie. Quoique la proportion d´hommes et de femmes qui travaillent dans l´agriculture est assez proche dans la région (77% chez les femmes et 82% chez les hommes) [17], les hommes sont toujours considérés comme étant ceux qui travaillent le plus et nécessiteraient de ce fait, un apport alimentaire plus important que les femmes. Cette discrimination liée au genre concerne plusieurs aspects de la vie quotidienne.

En effet, Madagascar est un pays où le genre est encore source d´inégalités diverses malgré que la situation y soit meilleure comparée à l´ensemble des pays sub-sahariens [19]. Une étude menée dans la région du Menabe montre que les femmes subissent des fortes pressions pour l´accouchement: elles ne doivent pas être un poids pour la famille ni recourir aux services de santé biomédicale tant que cela ne s´avère nécessaire. Ainsi, pour éviter d´accoucher de gros bébés, et risquer ainsi de subir une césarienne, elles restreignent leur alimentation pendant la grossesse et maintiennent des activités physiques jusqu´à leur accouchement. Ce qui pourrait aussi être source de malnutrition [10].

À Madagascar, être mère représente un privilège pour bénéficier d´un surplus de proximité affective et de confiance avec les enfants [20]. Notre étude rapporte pourtant que ce privilège peut avoir des impacts sur l´état nutritionnel des femmes qui devraient être les premières à se sacrifier pour les enfants. Partager sa part de nourriture à ses enfants si nécessaire, semble plus approprié et plus attendu d´une femme. Geste moins établi pour un homme. Par ailleurs, les hommes seraient considérés comme les plus actifs dans le ménage et de ce fait, auraient besoin d´un traitement particulier en matière d´alimentation. Cela pourrait déjà présager une inégalité entre femme et homme quant à l´accès aux mêmes nourritures. Cette différence de capabilité intrafamiliale d´accès aux aliments est régie par des normes socioculturelles et favorise la malnutrition chez les femmes [21].

Nos interviewés se disent être conscients de la gravité de la malnutrition. Pourtant, les individus qui travaillent dans le domaine de la santé affirment que le problème de malnutrition n´est pas considéré par la population comme un problème urgent ni grave. La malnutrition est tellement parlante que la population a fini par s´y adapter. Dans la région d´Amoron´i Mania, 41,6% des femmes en âge de procréer sont, en effet, en situation de malnutrition [8].

Du côté de la population, une volonté de changer les choses existe. Augmenter le rendement agricole ainsi que le revenu du ménage sont les principales solutions proposées. Ceci va dans le sens des interventions de lutte contre la malnutrition à Madagascar, visant à mobiliser les ressources et les produits disponibles [14]. Une adaptation de ces interventions aux possibilités locales et aux perceptions de la population (en prenant notamment en compte les disparités de genre à l´égard de l´alimentation) et un renforcement des interventions existantes pourraient accélérer l´obtention des résultats attendus.

Cette étude qualitative a été effectuée dans une zone à forte prévalence du problème de malnutrition et aussi, auprès des personnes vulnérables. Elle a permis d´appréhender et de comprendre en profondeur les connaissances et les perceptions des gens sur la malnutrition, des éléments qui pourraient aider à augmenter les effets des interventions de lutte contre ce fléau. Toutefois, ses limites résident dans le fait qu´elle ne peut prétendre représenter la situation générale existant dans le pays. Elle a été conduite dans une zone géographique limitée et auprès d´un échantillon restreint.

## Conclusion

La malnutrition renvoie généralement la population à une idée d´insuffisance alimentaire. La consommation quantitative de riz, aliment de base des malgaches, est toujours prise comme référence pour la définir et sa consommation en quantité suffisante suffirait pour se protéger de la malnutrition. Avec la pauvreté, les aspects culturels jouent également un rôle dans la malnutrition ainsi que dans la définition qu´on lui accorde. Une atteinte aux principales activités professionnelles et génératrices de revenu est considérée comme un facteur de vulnérabilité majeur. Certaines caractéristiques physiques (grossesse, âge d´adolescence, etc.) sont également reconnues comme des facteurs de vulnérabilité. Une conscience sur la gravité du problème est présente dans les esprits, une conscience ayant conduit parfois à une matérialisation de certaines ambitions individuelles pour le lutter. Cela constitue un paramètre encourageant pour les intervenants dans la lutte contre la malnutrition.

### Etat des connaissances sur le sujet

- Ampleur et principales causes de la malnutrition;
- Ampleur de l´insécurité alimentaire et ses conséquences dans les pays à faible et moyen revenu;
- Importance de l´implication de la population en tant qu´acteur dans la lutte contre la malnutrition.

### Contribution de notre étude à la connaissance

- Compréhension du problème de malnutrition par la population;
- Connaissances de la population sur les causes et la gravité de la malnutrition; perception des agents de santé sur facteurs socio-culturels qui favorisent la malnutrition;
- Mesures prises par la population pour lutter contre la malnutrition.

## Remerciements

Nous remercions Nestlé Nutrition Institute Africa (NNIA) qui a financé la collecte des données.
